# Loss of heterozygosity is related to *p53* mutations and smoking in lung cancer

**DOI:** 10.1054/bjoc.2000.1528

**Published:** 2001-01

**Authors:** S Zienolddiny, D Ryberg, M O Arab, V Skaug, A Haugen

**Affiliations:** Department of Toxicology, National Institute of Occupational Health, P.O. Box 8149 Dep., Oslo, N-0033, Norway

**Keywords:** non-small cell lung cancer, LOH, *p53* mutations, smoking, DNA-adducts

## Abstract

Carcinogenesis results from an accumulation of several genetic alterations. Mutations in the *p53* gene are frequent and occur at an early stage of lung carcinogenesis. Loss of multiple chromosomal regions is another genetic alteration frequently found in lung tumours. We have examined the association between *p53* mutations, loss of heterozygosity (LOH) at frequently deleted loci in lung cancer, and tobacco exposure in 165 tumours from non-small cell lung cancer (NSCLC) patients. A highly significant association between *p53* mutations and deletions on 3p, 5q, 9p, 11p and 17p was found. There was also a significant correlation between deletions at these loci. 86% of the tumours with concordant deletion in the 4 most involved loci (3p21, 5q11–13, 9p21 and 17p13) had *p53* mutations as compared to only 8% of the tumours without deletions at the corresponding loci (*P*< 0.0001). Data were also examined in relation to smoking status of the patients and histology of the tumours. The frequency of deletions was significantly higher among smokers as compared to non-smokers. This difference was significant for the 3p21.3 (*hMLH1* locus), 3p14.2 (*FHIT* locus), 5q11–13 (*hMSH3* locus) and 9p21 (*D9S157* locus). Tumours with deletions at the *hMLH1* locus had higher levels of hydrophobic DNA adducts. Deletions were more common in squamous cell carcinomas than in adenocarcinomas. Covariate analysis revealed that histological type and p53 mutations were significant and independent parameters for predicting LOH status at several loci. In the pathogenesis of NSCLC exposure to tobacco carcinogens in addition to clonal selection may be the driving force in these alterations. © 2001 Cancer Research Campaign http://www.bjcancer.com


*British Journal of Cancer* (2001) **84**(2), 226–231
© 2001 Cancer Research Campaign
doi: 10.1054/ bjoc.2000.1528, available online at http://www.idealibrary.com on IDEAL®

### Loss of heterozygosity is related to *p53* mutations and smoking in lung cancer

**S Zienolddiny, D Ryberg, MO Arab, V Skaug and A Haugen**

Department of Toxicology, National Institute of Occupational Health, P.O. Box 8149 Dep., N-0033 Oslo, Norway

**Summary** Carcinogenesis results from an accumulation of several genetic alterations. Mutations in the *p53* gene are frequent and occur at an early stage of lung carcinogenesis. Loss of multiple chromosomal regions is another genetic alteration frequently found in lung tumours. We have examined the association between *p53* mutations, loss of heterozygosity (LOH) at frequently deleted loci in lung cancer, and tobacco exposure in 165 tumours from non-small cell lung cancer (NSCLC) patients. A highly significant association between *p53* mutations and deletions on 3p, 5q, 9p, 11p and 17p was found. There was also a significant correlation between deletions at these loci. 86% of the tumours with concordant deletion in the 4 most involved loci (3p21, 5q11–13, 9p21 and 17p13) had *p53* mutations as compared to only 8% of the tumours without deletions at the corresponding loci ($P < 0.0001$). Data were also examined in relation to smoking status of the patients and histology of the tumours. The frequency of deletions was significantly higher among smokers as compared to non-smokers. This difference was significant for the 3p21.3 (*hMLH1* locus), 3p14.2 (*FHIT* locus), 5q11–13 (*hMSH3* locus) and 9p21 (*D9S157* locus). Tumours with deletions at the *hMLH1* locus had higher levels of hydrophobic DNA adducts. Deletions were more common in squamous cell carcinomas than in adenocarcinomas. Covariate analysis revealed that histological type and p53 mutations were significant and independent parameters for predicting LOH status at several loci. In the pathogenesis of NSCLC exposure to tobacco carcinogens in addition to clonal selection may be the driving force in these alterations. © 2001 Cancer Research Campaign http://www.bjcancer.com

**Keywords:** non-small cell lung cancer; LOH; *p53* mutations; smoking; DNA-adducts

Mutations in *p53* are among the most frequent genetic alterations detected in lung cancer and are acquired early in the development of the disease (Bennett et al, 1993; Bennett, 1995). In non-small cell lung cancer (NSCLC) about half of the tumours carry mutations in the *p53* gene (Takagi et al, 1998). The prevalent type of mutation is G:C → T:A transversion which is related to DNA adducts of benzo(a)pyrene from cigarette smoking (Greenblatt et al, 1994; Denissenko et al, 1996). *p53* plays an important role in preserving genomic stability through controlling the cell cycle checkpoints (Hartwell, 1992). Both human and mouse cells homozygous for *p53* mutations are genetically unstable and show high rates of homologous recombination (Livingstone et al, 1992; Yin et al, 1992; Mekeel et al, 1997). Other potential tumour suppressor genes are localized on chromosomes 3p, 5q, 9p and 11p (Kohno et al, 1999). It has been shown that deletions of chromosome 3p are early events in the development of the disease, and such changes are often observed in lung tumours (Wistuba et al, 1997). The *FHIT* gene at 3p14.2 has been shown to be a target of carcinogens in the tobacco smoke (Sozzi et al, 1997). The *hMLH1* gene maps to 3p21.3 and a high frequency of loss of heterozygosity (LOH) has been shown at this locus in lung cancer (Benachenhou et al, 1998). A concordant LOH of *hMLH1* locus and the *hMSH3* locus at 5q11–13 has been reported (Benachenhou et al, 1998). Mutations in the mismatch repair genes (MMR) are associated with a distinct molecular phenotype known as microsatellite instability (MSI) (Eshleman and Markowitz 1996). In lung cancer, the majority of studies have reported a low level (<5%) of MSI (Benachenhou et al, 1998). However, studies have shown that defects in DNA repair may be a predisposing factor in lung cancer (Wei and Spitz, 1997).

Lung cancer is characterized by multiple genetic changes (Gazdar, 1994). A relationship between *p53* mutations and 3p deletions has been reported in lung cancer (Horio et al, 1993; Burke et al, 1998; Kohno et al, 1999). The purpose of this study was to explore the association between tobacco exposure, *p53* mutations and concordant LOH at several loci harbouring potential tumour suppressor genes involved in lung cancer.

### MATERIALS AND METHODS

### Tissue samples

Surgically resected tumours and corresponding normal lung tissue were obtained from 165 lung cancer patients. The majority of tumours were at stage I according to international TNM staging. Each tissue was snap-frozen after surgery and stored at –80˚C. Prior to DNA isolation the tumour tissues were freeze-sectioned. 5–10 sections of each sample were stained with eosin and haematoxylin. Based on these slides, the tumours were trimmed to obtain more than 70% tumour cells. Histological classification of tumours was made according to guidelines made by the WHO Histological Typing of Lung Tumours (WHO, 1981). A questionnaire was filled out for each patient by the treating physician, including information on smoking habits. The characteristics of the lung cancer patients are summarized in Table 1.

### Analysis of LOH using microsatellite markers

17 polymorphic dinucleotide (CA) repeats representing regions with known DNA repair genes or tumour suppressor genes were

*Received 17 April 2000*
*Revised 1 September 2000*
*Accepted 14 September 2000*

*Correspondence to:* A Haugen

**Table 1** Characteristics of lung cancer patients

| Parameter | No. (%) | Mean (95% CI) |
|---|---|---|
| Total | 165 | |
| Male | 122 (73.9) | |
| Female | 43 (26.1) | |
| Histological subtype | | |
| Adenocarcinoma | 69 (41.8) | |
| Squamous cell carcinoma | 68 (41.2) | |
| Other[a] | 28 (17.0) | |
| Age | | 62.1 (60.4–63.9) |
| Smoking history | | |
| Smokers | 147 (89.1) | |
| Cigarettes per day | | 15.6 (14.4–16.7) |
| Pack-years | | 30.7 (28.1–33.4) |
| Years of smoking | | 39.6 (37.4–41.7) |
| Non-smokers[b] | 15 (9.1) | |
| Unknown | 3 (1.8) | |

CI, confidence interval; [a]Other includes unknown, large cell and small cell (2 cases) carcinomas; [b]Not smoked for the past 20 years.

analysed. The markers D2S123 and D2S391 are linked to *hMSH2-hMSH6* mismatch repair genes on chromosome 2p16–21. D3S1300 is mapped within the *FHIT* gene (3p14.2), D3S1611 is internal to the *hMLH1* gene (3p21) and D3S1612 is closely linked to it. The *hMSH3* gene is located near D5S431 marker at 5q11–13. *hPMS2* is mapped near D7S531 locus (7p22). The markers T*P53* and D17S786 are linked to the *p53* gene. The other markers represent chromosomal regions often deleted in lung cancer. The PCR methods and sequences of the primers except for D2S123 and D11S4181 are previously published (Lindstedt et al, 1999). The primer sequences for the 2 markers are: D2S123, 5′-GCCTGCCTTTAACAGTGCTA-3′, 5′-AGGGGACTTTCCACCTATG-3′; D11S4181,5′-AGAGGC-AGGAGAATCACTTG3′,5′-CACTAAACATCCAGCTCAA-A-3′. The map positions of the markers are based on the Human Genetic Map available in various databases. DNA samples were analysed for LOH by multiplex PCR and capillary electrophoresis as described (Canzian et al, 1996). To facilitate multiplexing, the size of the amplified product and the annealing temperatures were considered. The oligonucleotides were labelled fluorescently with one of the 3 dyes (6-FAM, TET, HEX; Applied Biosystems) and the fourth dye TAMRA was reserved for the size standard. After collection, data were analysed using the Gene Scan software (Applied Biosystems) and allelic imbalance was determined and LOH was defined as at least 50% loss of one allele in the tumour tissue after correction for the non-tumour tissue.

### Determination of PAH-DNA adducts

The PAH-DNA adducts were analysed by the $^{32}$P-postlabelling method as described previously and published (Mollerup et al, 1999).

### Analysis of *p53* mutations

The *p53* mutation analysis was performed using a modified single-strand conformation polymorphism (SSCP) procedure and direct sequencing as described (Kure et al, 1996; Skaug et al, 2000).

### Statistical methods

The hypothesis of independence in LOH at the 4 most involved loci was tested using likelihood ratio test with correction for continuity. Logistic regression analysis (SPSS v. 10) was used to identify important variables predicting LOH and to calculate adjusted *P* values. The following variables were included in the model: *p53* mutational status, smoking status, gender, age and histological types. *p53* mutational status and histological types were the only significant parameters predicting LOH status at most of the loci. Since the number of non-smoker patients was low in this study, the association between smoking status and LOH status was analysed using Fishers exact test. The relationship between PAH adduct levels and LOH status was analysed by Wilcoxon rank sum test. The StatXact version 4 program was used for trend analysis.

### RESULTS

Paired tumour/normal DNA samples from 165 NSCLC patients (Table 1) were examined for allelic imbalance at 17 polymorphic microsatellite markers in 7 different chromosomes. High LOH frequencies (about 50%) were observed with 3p, 5q, 9p and 17p markers, moderate LOH at 11p15, and low frequencies at 7p and 2p (Table 2). There was a significant correlation between deletions in markers at 3p, 5q, 9p, 11p and 17p loci (data not shown). The concordant LOH at the 4 most deleted loci 3p21, 5q11–13, 9p21 and 17p13 based on data from the markers D3S1611-D3S1612, D5S431, D9S157 and T*P53*-D17S786, respectively, was further examined. 83 cases were informative for all these 4 loci; 14 cases were deleted at all 4 loci, 21 deleted at 3 loci, 14 at 2 loci, 9 at one locus, and 25 cases had retained heterozygosity at all 4 loci. The corresponding expected frequencies based on independence would be 4.5, 19.3, 31.1, 22.2 and 5.9 cases. This difference in the observed and expected frequencies of concordant LOH at the 4 loci was statistically significant and the possibility that these deletions may have occurred independently should be rejected ($P = 0.03 \times 10^{-5}$, likelihood ratio test with correction for continuity). Similar results were obtained using other markers at 3p, 5q and 17p. Of 115 cases informative for *hMLH1* (D3S1611-D3S1612) and *hMSH3* (D5S431), 47 (41%) were deleted

**Table 2** Frequency of LOH at different microsatellite loci in lung cancer patients

| Microsatellite marker | Informative cases *n* (%) | LOH[a] *n* (%) |
|---|---|---|
| D2S123 | 127 (77.4) | 19 (15.0) |
| D2S391 | 123 (75.0) | 15 (12.2) |
| D3S1611 | 109 (66.1) | 51 (46.8) |
| D3S1612 | 117 (71.3) | 55 (47.0) |
| D3S966 | 128 (77.6) | 61 (47.7) |
| D3S1289 | 144 (87.3) | 66 (45.8) |
| D3S1300 | 136 (82.4) | 64 (47.1) |
| D5S1968 | 126 (76.8) | 56 (44.4) |
| D5S2089 | 134 (81.7) | 56 (41.8) |
| D5S431 | 131 (79.4) | 60 (45.8) |
| D5S495 | 109 (66.5) | 45 (41.3) |
| D7S531 | 134 (81.2) | 18 (13.4) |
| D9S157 | 136 (82.9) | 65 (47.8) |
| D11S4181 | 124 (79.5) | 45 (36.2) |
| D17S799 | 123 (75.0) | 61 (49.6) |
| D17S786 | 122 (73.9) | 62 (50.8) |
| TP53 | 73 (44.5) | 33 (45.2) |

[a]LOH = no. of cases with deletions/no. of informative cases.

at both loci. However, this was not specific for these 2 loci since similar frequencies were found at other loci investigated.

The *p53* mutations were found in 86 (52%) of the 165 tumours analysed (Kure et al, 1996; Skaug et al, 2000). Using logistic regression and adjusting for other variables there was a significant association between the presence of *p53* mutations and occurrence of LOH at 3p, 5q, 9p, 11p and 17p (Table 3). Similar results were also obtained when smoking status was replaced by smoking years or pack-years in the logistic models. Of the 14 cases with concordant LOH at the 4 most deleted regions (see above), 12 (85.7%) had mutations in the *p53* gene. The corresponding frequency of mutations in the 25 tumours without LOH in these loci was only 2 (8%) ($P < 0.0001$, Fishers exact test). There was also a significant correlation between the frequency of *p53* mutations and the number of loci deleted (Figure 1, $P < 0.0001$ trend test). Of 47 cases deleted in both *hMLH1* and *hMSH3* loci, 37 (79%) had *p53* mutations whereas only 11 (22%) of the 49 cases without LOH were mutated in *p53* ($P < 0.0001$, Fisher exact test). The LOH data were also analysed with respect to *p53* mutational types and positions without any particular findings.

The frequency of LOH was significantly lower in non-smokers as compared to smokers for the *hMLH1* locus, D3S1300 (*FHIT*), D5S431 (*hMSH3*), and D9S157 using table analysis and Fishers exact test (Table 4). Using logistic regression and adjusting for *p53* mutational status, histological types, age and gender only the D9S157 marker gave significant association. Since the number of non-smokers is low and *p53* mutations are nearly absent among non-smoking patients, the last method is less reliable. Tumours deleted in the *hMLH1* locus showed higher PAH-DNA adduct levels ($P = 0.078$, Wilcoxon rank test) and there were more tumours with LOH in this locus in the upper adduct tertile (≥12.36 adducts/$10^8$ nucleotides) than in the lower tertile group (≤6.67 adducts/$10^8$ nucleotides, $P = 0.06$, Fisher's exact test). Other loci on 3p showed similar tendency, but the statistics were weaker.

The frequency of LOH was significantly higher in squamous cell carcinomas compared to adenocarcinomas for several loci after adjusting for *p53* mutational status, smoking status, gender and age (Table 5). Similar results were seen when smoking years and pack-years replaced smoking status in the logistic models. Among the 14 tumours with LOH at all the 4 most involved loci, 9 (64.3%) were squamous cell carcinomas and 3 (21.4%) were adenocarcinomas.

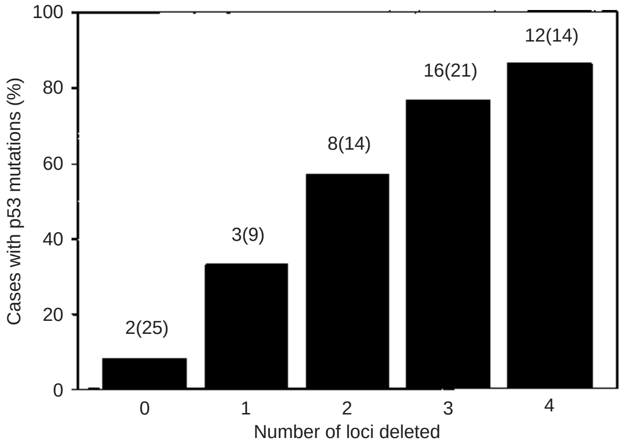

**Figure 1** Correlation between incidence of *p53* mutations and number of loci deleted in tumours informative for all the 4 loci, 3p21, 5q11–13, 9p21 and 17p13. Figures in ( ) indicate no. of informative cases. Trend test: $P = 0.0001$

### DISCUSSION

Lung tumours harbour several genetic abnormalities including *p53* mutations and loss of several chromosomal regions. In this study we have examined possible interactions between several genetic alterations in the tumours, particularly at loci with potential tumour suppressor genes.

Analysis of the sequence of abnormalities in lung cancer has shown that deletions in the 3p region are early events and precede deletions in 9p, 17p (*TP53* locus) and 5q (Wistuba et al, 1999).

**Table 3** Frequencies of LOH at individual locus in relation to mutational status of the *p53* gene

| ***p53* gene status** | **2p16–21 (D2S123–D2S391)[a]** | **3p21.3 (D31611–D3S1612)[a]** | **3p14.2 (D3S1300)** | **5q11–13 (D5S431)** | **5q21 (D5S495)** | **7p22 (D7S531)** | **9p21 (D9S157)** | **11p15 (D11S4181)** | **17p13 (TP53–D17S786)[a]** |
|---|---|---|---|---|---|---|---|---|---|
| Mutated | 11/72 | 52/76 | 44/71 | 44/66 | 31/56 | 10/72 | 48/74 | 34/65 | 51/75 |
| Normal | 5/71 | 20/69 | 20/65 | 16/65 | 14/53 | 8/62 | 17/62 | 11/59 | 19/67 |
| $P^b$ | NS | 0.001 | 0.03 | 0.001 | 0.04 | NS | 0.001 | 0.003 | 0.002 |

[a]LOH at one or both markers. [b]*P*-values based on logistic regression adjusting for smoking status, gender, age and histological types. NS – not significant.

**Table 4** Frequency of LOH in smokers and non-smokers

| **Chromosome** | **2p16–21 (D2S123–D2S391)[a]** | **3p21.3 (D31611–D3S1612)[a]** | **3p14.2 (D3S1300)** | **5q11–13 (D5S431)** | **5q21 (D5S495)** | **7p22 (D7S531)** | **9p21 (D9S157)** | **11p15 (D11S4181)** | **7p13 (TP53–D17S786)[a]** |
|---|---|---|---|---|---|---|---|---|---|
| Smokers[b] | 25/134 | 70/129 | 63/124 | 59/115 | 43/99 | 16/121 | 63/121 | 38/105 | 65/129 |
| Non-smokers[b] | 0/15 | 2/14 | 1/10 | 1/12 | 2/7 | 2/11 | 1/14 | 3/14 | 4/11 |
| P (Fishers exact test) | 0.08 | 0.005 | 0.02 | 0.005 | NS | NS | 0.001 | NS | NS |

[a]LOH at one or both markers; NS – not significant.

*© 2001 Cancer Research Campaign*

**Table 5** Frequencies of LOH at individual locus in relation to the two major histological types

| Histological subtype | 2p16–21 (D2S123–D2S391)[a] | 3p21.3 (D31611–D3S1612)[a] | 3p14.2 (D3S1300) | 5q11–13 (D5S431) | 5q21 (D5S495) | 7p22 (D7S531) | 9p21 (D9S157) | 11p15 (D11S4181) | 17p13 (TP53–D17S786)[a] |
|---|---|---|---|---|---|---|---|---|---|
| Adenocarcinoma | 8/60 | 22/59 | 20/53 | 17/55 | 11/46 | 6/56 | 26/58 | 19/55 | 27/60 |
| Squamous cell | 3/61 | 40/62 | 35/62 | 33/53 | 23/42 | 8/54 | 29/53 | 19/46 | 34/60 |
| $P^b$ | NS | 0.05 | 0.08 | 0.03 | 0.02 | NS | NS | NS | 0.04 |

[a]LOH at one or both markers. [b]*P*-values based on logistic regression adjusting for p53 mutational status, smoking status, age and gender. NS – not significant.

Further deletion mapping of the 3p region has revealed chromosomal regions 3p21 and 3p14 to be most frequently deleted in many cancer types including NSCLC (Mitsudomi et al, 1996; Todd et al, 1997; Benachenhou et al, 1998; Nelson et al, 1998). The relationship between allelic losses at the various loci, may indicate an interaction between various gene(s) in these loci during the process of clonal selection.

The high frequency of deletions found at the *hMLH1* and *hMSH3* loci may indicate a possible role for these loci in NSCLC. A possible interdependence of LOH at these loci has been indicated (Benachenhou et al, 1998). This study confirmed concomitant LOH in cases informative for both loci. However, a similar concomitant LOH frequency was also found when other frequently affected regions were investigated. Similar LOH frequencies were also observed for other markers within the 3p region and 5q region indicating an interaction between several genes, not specifically on 3p21 and 5q11–13. The unexpectedly high number of tumours without apparent LOH at the 4 most involved loci at 3p21, 5q11–13, 9p21 and 17p13 (25 cases against the expected 5.9 cases) may indicate that specific mechanisms are involved, resulting in frequent deletions in all these loci. DNA repair genes like *hMLH1* and *hMSH3*, and the *p53* gene may be involved in these mechanisms. Deficiencies in MMR genes are associated with several cancers often resulting in MSI in tumours (Lengauer et al, 1997). MSI in lung cancer occurs at a low frequency (Benachenhou et al, 1998). However, MMR proteins are involved in a variety of other cellular processes such as homologous recombination (de Wind et al, 1995), mediation of the G2 checkpoint (Meyers et al, 1997; Davis et al, 1998), the signalling pathway and transcription-coupled nucleotide excision repair (Mellon et al, 1996). Gene-dosage effects and reduced expression of MMR proteins have been suggested as risk factors (Wei et al, 1996, 1998; Benachenhou et al, 1998). Other DNA repair genes such as *hRad51* and *hOGG1* may also be involved (Buchhop et al, 1997; Chevillard et al, 1998). Several genes localized at the other frequently deleted loci such as *FHIT* (3p14.2), *APC* (5q21), *p16* (9p21) and other genes at 17p may also interact with each other. Correlations between *p53* mutations and LOH at *FHIT* locus and 9p have also been reported in lung tumours by others (Marchetti et al, 1998; Kohno et al, 1999). The low rate of allelic losses (12–15%) affecting *hMSH2-hMSH6* (2p21–16) and *hPMS2* (7p22) loci is consistent with previous reports and may reflect the base line LOH in cancer cells (Benachenhou et al, 1998, 1999). This may also indicate that deletion may not be an inactivating mechanism for these genes or these genes may play a minor role in lung cancer.

The *p53* gene was mutated in 52% of NSCLC cases. This frequency is consistent with the average incidence reported in NSCLC (Greenblatt et al, 1994; Hollstein et al, 1994). A significantly higher number of tumours with LOH had *p53* mutations compared to tumours without LOH. Logistic regression analysis revealed that *p53* mutational status was the most significant variable for explaining LOH at most of the examined loci. The highly significant trend ($P < 0.0001$, trend test) toward increased frequency of *p53* mutations with the higher number of loci deleted, may further support a relationship between these two types of genetic alterations. Among samples with concordant LOH at the 4 most deleted loci all 12 samples with *p53* mutations had mutations in reported hotspot codons (Walker et al, 1999). Hotspot somatic mutations in cancers may represent protein alterations which provide a selective growth advantage to the cell. Genomic instability in a subset of non-smoking lung cancer patients has been related to mutations in the *p53* gene (Pellegrini et al, 1999). Genomic instability induced by *p53* may arise through abnormal cell cycle check points (Hartwell, 1992). Considering the involvement of both *p53* and MMR genes in cell cycle, an interaction between them in maintaining genomic stability may seem reasonable. However, no correlation between MSI and *p53* mutations has been found in NSCLC (Caligo et al, 1998). Other pathways such as recombination have also been reported to be involved in induction of LOH in normal human cells (Gupta et al, 1997) and loss of both alleles of *p53* in a primary human glioblastoma tumour and cell line (Albertoni et al, 1998).

For all loci examined (except 7p), there was a clear trend of higher occurrence of LOH in smokers than non-smokers. While number of cases with LOH was significantly lower among non-smokers in both regions of 3p (3p 14.2 and 3p21), this was only significant for the 5q11–13 (*hMSH*3 locus) locus on chromosome 5q. Despite the low number of non-smoking cases, this may indicate a possible association between smoking and occurrence of LOH. The differences in association of LOH with the smoking status may suggest that the genetic loci may differ in their sensitivity to the mutagenic effects of carcinogens. Associations between cigarette smoking and *FHIT* gene (3p14.2) alterations have been reported (Sozzi et al, 1997; Tomizawa et al, 1998). Deletions within the LOH11B locus on 11p have also been associated with high cigarette consumption (Schreiber et al, 1997). Significantly higher *p53* mutations in tumours and PAH-DNA adduct levels in lung tissue are observed in smoking than non-smoking lung cancer patients (Ryberg et al, 1994a, 1994b). The higher frequency of tumours with LOH in 3p21.3 locus in the upper adduct tertile may indicate a higher sensitivity of this locus to tobacco carcinogens. An association of increased 3p21 LOH with increasing PAH-DNA adducts in squamous cell carcinomas of the lung has recently been reported (Hirao et al, 2000) and Wu et al, (1998) demonstrated a dose–response relationship between BPDE exposure and 3p21.3 (*hMLH1* region) aberrations in lymphocytes from lung cancer patients.

Adenocarcinoma and squamous cell carcinoma are the two major histological types found in NSCLC. This study showed that

*© 2001 Cancer Research Campaign* 

allelic deletions were more frequent in squamous cell carcinomas for most loci. After adjusting for *p53* mutational status, smoking, gender and age the difference between the two subtypes became significant for the 3p, 5q and 17p regions. Different pattern of genetic changes have previously been reported for the 2 histological types (Sato et al, 1994). We have also found a significantly higher frequency of *p53* mutations in squamous cell carcinomas than adenocarcinomas (Kure et al, 1996; Skaug et al, 2000). The development of different histological types of lung tumours has been suggested to reflect a dose effect of tobacco carcinogens. More centrally located cells of the airways giving rise to squamous cell carcinomas are exposed to a greater carcinogen concentration than more distal cells giving rise to the more peripherally located adenocarcinomas (Burke et al, 1998).

Our data indicate that LOH at 3p, 5q, 9p, 11p and 17p regions, often affected in NSCLC, are interdependent and highly associated with mutated *p53* gene. Compounds in the tobacco smoke in addition to clonal selection may be the driving force in these alterations.

### ACKNOWLEDGEMENTS

We thank Ms Rita Bæra for excellent technical assistance and Dr Lodve Stangeland for providing lung tissue and patient data. This project was supported by The Norwegian Research Council, The Norwegian Cancer Society and EU grant ENV4-CT97-0469.

### REFERENCES

Albertoni M, Daub DM, Arden KC, Viars CS, Powell C and Van Meir EG (1998) Genetic instability leads to loss of both *p53* alleles in a human glioblastoma. *Oncogene* **16**: 321–326

Benachenhou N, Guiral S, Gorska-Flipot I, Labuda D and Sinnett D (1998) High resolution deletion mapping reveals frequent allelic losses at the DNA mismatch repair loci *hMLH1* and *hMSH3* in non-small cell lung cancer. *Int J Cancer* **77**: 173–180

Benachenhou N, Guiral S, Gorska-Flipot I, Labuda D and Sinnett D (1999) Frequent loss of heterozygosity at the DNA mismatch-repair loci *hMLH1* and *hMSH3* in sporadic breast cancer. *Br J Cancer* **79**: 1012–1017

Bennett WP (1995) *p53* alterations in progenitor lesions of the bronchus, esophagus, oral cavity, and colon. *Cancer Detect Prev* **19**: 503–511

Bennett WP, Colby TV, Travis WD, Borkowski A, Jones RT, Lane DP, Metcalf RA, Samet JM, Takeshima Y and Gu JR (1993) *p53* protein accumulates frequently in early bronchial neoplasia. *Cancer Res* **53**: 4817–4822

Buchhop S, Gibson MK, Wang XW, Wagner P, Sturzbecher HW and Harris CC (1997) Interaction of *p53* with the human Rad51 protein. *Nucleic Acids Res* **25**: 3868–3874

Burke L, Khan MA, Freedman AN, Gemma A, Rusin M, Guinee DG, Bennett WP, Caporaso NE, Fleming MV, Travis WD, Colby TV, Trastek V, Pairolero PC, Tazelaar HD, Midthun DE, Liotta LA and Harris CC (1998) Allelic deletion analysis of the *FHIT* gene predicts poor survival in non-small cell lung cancer. *Cancer Res* **58**: 2533–2536

Caligo MA, Ghimenti C, Marchetti A, Lonobile A, Buttitta F, Pellegrini S and Bevilacqua G (1998) Microsatellite alterations and *p53*, *TGF*betaRII, *IGFIIR* and *BAX* mutations in sporadic non-small-cell lung cancer. *Int J Cancer* **78**: 606–609

Canzian F, Salovaara R, Hemminki A, Kristo P, Chadwick RB, Aaltonen LA and de la Chapelle A (1996) Semiautomated assessment of loss of heterozygosity and replication error in tumors. *Cancer Res* **56**: 3331–3337

Chevillard S, Radicella JP, Levalois C, Lebeau J, Poupon MF, Oudard S, Dutrillaux B and Boiteux S (1998) Mutations in *OGG1*, a gene involved in the repair of oxidative DNA damage, are found in human lung and kidney tumours. *Oncogene* **16**: 3083–3086

Davis TW, Wilson-Van PC, Meyers M, Kunugi KA, Cuthill S, Reznikoff C, Garces C, Boland CR, Kinsella TJ, Fishel R and Boothman DA (1998) Defective expression of the DNA mismatch repair protein, MLH1, alters G2-M cell cycle checkpoint arrest following ionizing radiation. *Cancer Res* **58**: 767–778

de Wind N, Dekker M, Berns A, Radman M and te RH (1995) Inactivation of the mouse Msh2 gene results in mismatch repair deficiency, methylation tolerance, hyperrecombination, and predisposition to cancer. *Cell* **82**: 321–330

Denissenko MF, Pao A, Tang M and Pfeifer GP (1996) Preferential formation of benzo[a]pyrene adducts at lung cancer mutational hotspots in *p53*. *Science* **274**: 430–432

Eshleman JR and Markowitz SD (1996) Mismatch repair defects in human carcinogenesis. *Hum Mol Genet* **5**: 1489–1494

Gazdar AF (1994) The molecular and cellular basis of human lung cancer. *Anticancer Res* **14**: 261–267

Greenblatt MS, Bennett WP, Hollstein M and Harris CC (1994) Mutations in the *p53* tumor suppressor gene: clues to cancer etiology and molecular pathogenesis. *Cancer Res* **54**: 4855–4878

Gupta PK, Sahota A, Boyadjiev SA, Bye S, Shao C, O'Neill JP, Hunter TC, Albertini RJ, Stambrook PJ and Tischfield JA (1997) High frequency in vivo loss of heterozygosity is primarily a consequence of mitotic recombination. *Cancer Res* **57**: 1188–1193

Hartwell L (1992) Defects in a cell cycle checkpoint may be responsible for the genomic instability of cancer cells. *Cell* **71**: 543–546

Hirao T, Nelson HH, Ashok TDS, Wiencke JK, Zu Z-f, Gunn L, Wain JC, Mark EW, Christiani DC and Kelsey KT (2000) Early smoking and increased DNA adduct burden predict LOH at 3p21 in NSCLC. *Proc Am Ass Cancer Res* **41**: 321 (Abstract)

Hollstein M, Rice K, Greenblatt MS, Soussi T, Fuchs R, Sorlie T, Hovig E, Smith-Sorensen B, Montesano R and Harris CC (1994) Database of *p53* gene somatic mutations in human tumors and cell lines. *Nucleic Acids Res* **22**: 3551–3555

Horio Y, Takahashi T, Kuroishi T, Hibi K, Suyama M, Niimi T, Shimokata K, Yamakawa K, Nakamura Y and Ueda R (1993) Prognostic significance of *p53* mutations and 3p deletions in primary resected non-small cell lung cancer. *Cancer Res* **53**: 1–4

Kohno H, Hiroshima K, Toyozaki T, Fujisawa T and Ohwada H (1999) *p53* mutation and allelic loss of chromosome 3p, 9p of preneoplastic lesions in patients with nonsmall cell lung carcinoma. *Cancer* **85**: 341–347

Kohno T, Yokota J (1999) How many tumor suppressor genes are involved in human lung carcinogenesis? *Carcinogenesis* **20**: 1403–1410

Kure EH, Ryberg D, Hewer A, Phillips DH, Skaug V, Baera R and Haugen A (1996) *p53* mutations in lung tumours: relationship to gender and lung DNA adduct levels. *Carcinogenesis* **17**: 2201–2205

Lengauer C, Kinzler KW and Vogelstein B (1997) Genetic instability in colorectal cancers. *Nature* **386**: 623–627

Lindstedt BA, Ryberg D, Zienolddiny S, Khan H and Haugen A (1999) Hras 1 VNTR alleles as susceptibility markers for lung cancer: relationship to microsatellite instability in tumors. *Anticancer Res* **19**: 5523–5527

Livingstone LR, White A, Sprouse J, Livanos E, Jacks T and Tlsty TD (1992) Altered cell cycle arrest and gene amplification potential accompany loss of wild-type *p53*. *Cell* **70**: 923–935

Marchetti A, Pellegrini S, Sozzi G, Bertacca G, Gaeta P, Buttitta F, Carnicelli V, Griseri P, Chella A, Angeletti CA, Pierotti M and Bevilacqua G (1998) Genetic analysis of lung tumours of non-smoking subjects: *p53* gene mutations are constantly associated with loss of heterozygosity at the *FHIT* locus. *Br J Cancer* **78**: 73–78

Mekeel KL, Tang W, Kachnic LA, Luo CM, DeFrank JS and Powell SN (1997) Inactivation of *p53* results in high rates of homologous recombination. *Oncogene* **14**: 1847–1857

Mellon I, Rajpal DK, Koi M, Boland CR and Champe GN (1996) Transcription-coupled repair deficiency and mutations in human mismatch repair genes. *Science* **272**: 557–560

Meyers M, Theodosiou M, Acharya S, Odegaard E, Wilson T, Lewis JE, Davis TW, Wilson-Van PC, Fishel R and Boothman DA (1997) Cell cycle regulation of the human DNA mismatch repair genes *hMSH2*, *hMLH1*, and *hPMS2*. *Cancer Res* **57**: 206–208

Mitsudomi T, Oyama T, Nishida K, Ogami A, Osaki T, Sugio K, Yasumoto K, Sugimachi K and Gazdar AF (1996) Loss of heterozygosity at 3p in non-small cell lung cancer and its prognostic implication. *Clin Cancer Res* **2**: 1185–1189

Mollerup S, Ryberg D, Hewer A, Phillips DH and Haugen A (1999) Sex differences in lung CYP1A1 expression and DNA adduct levels among lung cancer patients. *Cancer Res* **59**: 3317–3320

Nelson HH, Wiencke JK, Gunn L, Wain JC, Christiani DC and Kelsey KT (1998) Chromosome 3p14 alterations in lung cancer: evidence that *FHIT* exon deletion is a target of tobacco carcinogens and asbestos. *Cancer Res* **58**: 1804–1807

© 2001 Cancer Research Campaign

Pellegrini S, Bertacca G, Buttitta F, Bevilacqua G and Marchetti A (1999) Lung tumours from non-smoking subjects: A *p53*-related genetic instability in a subset of cases. *Int J Mol Med* **4**: 419–424

Ryberg D, Hewer A, Phillips DH and Haugen A (1994a) Different susceptibility to smoking-induced DNA damage among male and female lung cancer patients. *Cancer Res* **54**: 5801–5803

Ryberg D, Kure E, Lystad S, Skaug V, Stangeland L, Mercy I, Borresen AL and Haugen A (1994b) *p53* mutations in lung tumors: relationship to putative susceptibility markers for cancer. *Cancer Res* **54**: 1551–1555

Sato S, Nakamura Y and Tsuchiya E (1994) Difference of allelotype between squamous cell carcinoma and adenocarcinoma of the lung. *Cancer Res* **54**: 5652–5655

Schreiber G, Fong KM, Peterson B, Johnson BE, O'Briant KC and Bepler G (1997) Smoking, gender, and survival association with allele loss for the LOH11B lung cancer region on chromosome 11. *Cancer Epidemiol Biomarkers Prev* **6**: 315–319

Skaug V, Ryberg D, Kure EH, Arab MO, Stangeland L, Myking AO and Haugen A (2000) *p53* mutations in defined structural and functional domains are related to poor clinical outcome in non-small cell lung cancer patients. *Clin Cancer Res* **6**: 1031–1037

Sozzi G, Sard L, De Gregorio L, Marchetti A, Musso K, Buttitta F, Tornielli S, Pellegrini S, Veronese ML, Manenti G, Incarbone M, Chella A, Angeletti CA, Pastorino U, Huebner K, Bevilaqua G, Pilotti S, Croce CM and Pierotti MA (1997) Association between cigarette smoking and *FHIT* gene alterations in lung cancer. *Cancer Res* **57**: 2121–2123

Takagi Y, Osada H, Kuroishi T, Mitsudomi T, Kondo M, Niimi T, Saji S, Gazdar AF, Takahashi T and Minna JD (1998) *p53* mutations in non-small-cell lung cancers occurring in individuals without a past history of active smoking. *Br J Cancer* **77**: 1568–1572

Todd S, Franklin WA, Varella-Garcia M, Kennedy T, Hilliker CEJ, Hahner L, Anderson M, Wiest JS, Drabkin HA and Gemmill RM (1997) Homozygous deletions of human chromosome 3p in lung tumors. *Cancer Res* **57**: 1344–1352

Tomizawa Y, Nakajima T, Kohno T, Saito R, Yamaguchi N and Yokota J (1998) Clinicopathological significance of Fhit protein expression in stage I non-small cell lung carcinoma. *Cancer Res* **58**: 5478–5483

Walker DR, Bond JP, Tarone RE, Harris CC, Makalowski W, Boguski MS and Greenblatt MS (1999) Evolutionary conservation and somatic mutation hotspot maps of *p53*: correlation with *p53* protein structural and functional features. *Oncogene* **18**: 211–218

Wei Q and Spitz MR (1997) The role of DNA repair capacity in susceptibility to lung cancer: a review. *Cancer Metastasis Rev* **16**: 295–307

Wei Q, Cheng L, Hong WK and Spitz MR (1996) Reduced DNA repair capacity in lung cancer patients. *Cancer Res* **56**: 4103–4107

Wei Q, Eicher SA, Guan Y, Cheng L, Xu J, Young LN, Saunders KC, Jiang H, Hong WK, Spitz MR and Strom SS (1998) Reduced expression of *hMLH1* and hGTBP/hMSH6: a risk factor for head and neck cancer. *Cancer Epidemiol Biomakers Prev* **7**: 309–314

Wistuba II, Montellano FD, Milchgrub S, Virmani AK, Behrens C, Chen H, Ahmadian M, Nowak JA, Muller C, Minna JD and Gazdar AF (1997) Deletions of chromosome 3p are frequent and early events in the pathogenesis of uterine cervical carcinoma. *Cancer Res* **57**: 3154–3158

Wistuba II, Behrens C, Milchgrub S, Bryant D, Hung J, Minna JD and Gazdar AF (1999) Sequential molecular abnormalities are involved in the multistage development of squamous cell lung carcinoma. *Oncogene* **18**: 643–650

Wu X, Zhao Y, Honn SE, Tomlinson GE, Minna JD, Hong WK and Spitz MR (1998) Benzo[a]pyrene diol epoxide-induced 3p21.3 aberrations and genetic predisposition to lung cancer. *Cancer Res* **58**: 1605–1608

Yin Y, Tainsky MA, Bischoff FZ, Strong LC and Wahl GM (1992) Wild-type *p53* restores cell cycle control and inhibits gene amplification in cells with mutant *p53* alleles. *Cell* **70**: 937–948

*© 2001 Cancer Research Campaign*
